# Role of Autologous Haematopoietic Transplantation in Leukaemias: When to Consider It in 2026

**DOI:** 10.3390/hematolrep18040044

**Published:** 2026-06-29

**Authors:** Miklós Udvardy, Lajos Gergely, Róbert Szász, Gyula Reményi, László Imre Pinczés, Árpád Illés

**Affiliations:** Division of Haematology, Department of Internal Medicine, Faculty of Medicine, University of Debrecen, Member of ERN-EuroBlood-NET, H-4032 Debrecen, Hungary; lgergely@med.unideb.hu (L.G.); szaszr@med.unideb.hu (R.S.); gremenyi@med.unideb.hu (G.R.); pinczes.laszlo.imre@med.unideb.hu (L.I.P.); illes.arpad@med.unideb.hu (Á.I.)

**Keywords:** autologous transplantation, acute myeloid leukaemia, plasma cell leukaemia, innovative approaches to improve results in autologous settings

## Abstract

**Background:** This review aims to provide a comprehensive and practical overview of the evolving role of autologous transplantation in leukaemias, a strategy that was once largely abandoned but has recently regained interest in selected clinical settings. **Methods:** We reviewed the historical development of autologous transplantation in acute leukaemias, including the early period during which autologous transplantation was considered inferior to allogeneic approaches because of limited graft purification techniques and the inability to induce effective graft-versus-leukaemia (GVL)-like immune responses. We further summarise more recent experimental strategies aimed at improving stem cell purification and enhancing anti-leukaemic immune activity in autologous settings. In addition, we discuss how advances in measurable residual disease (MRD) assessment and molecular risk stratification have contributed to the renewed interest in autologous transplantation in selected subgroups of leukaemia patients. **Results:** This review identifies clinical situations in which autologous transplantation remains an important therapeutic option, including plasma cell leukaemia, where it continues to represent a standard first-line approach. We also discuss well-defined patient subgroups, particularly selected AML subtypes with intermediate-risk molecular profiles and acute promyelocytic leukaemia (APL) in second remission, in which outcomes following autologous transplantation may be comparable to, or occasionally superior to, those achieved with allogeneic transplantation. In contrast, autologous transplantation currently plays only a limited role in diseases such as chronic lymphocytic leukaemia (CLL) and chronic myeloid leukaemia (CML). Although attempts to induce potent anti-leukaemic immune effects in autologous settings have so far shown limited clinical efficacy, several emerging strategies appear promising and may further expand the role of autologous transplantation, particularly in elderly or frail patients. **Discussion:** Overall, current molecular and MRD-based risk stratification strategies, together with emerging immunological and graft-manipulation approaches, may redefine the role of autologous transplantation as a personalised therapeutic option in selected subgroups of leukaemia patients.

## 1. Introduction

### 1.1. History

Decades ago, autologous stem cell transplantation was considered a powerful alternative to allogeneic transplantation in acute myeloid leukaemia (AML), sometimes replacing costly post-induction high-dose cytosine arabinoside-based therapies. This concept was largely driven by the pioneering work of Gorin, Burnett, and Stuart [1,2]. Three major factors supported the use of autologous transplantation at that time. First, the availability of suitable allogeneic donors was limited. Second, allogeneic transplantation was associated with a relatively high early transplant-related mortality (TRM). Third, post-induction therapies for acute leukaemias were associated with substantial costs.

Early recommendations for autologous transplantation in AML focused on both conditioning regimens and the optimal timing of transplantation. Initially, conditioning consisted mainly of total body irradiation (TBI) plus cyclophosphamide or busulfan-cyclophosphamide (Bu-Cy). In contrast, later regimens included fludarabine-melphalan (Flu-Mel) and busulfan-melphalan (Bu-Mel), among others. At that time, autologous transplantation was generally recommended not to be performed too early after post-induction therapy, although this concept later changed considerably [1,2,3].

Over time, donor availability improved significantly, primarily due to the introduction of umbilical cord blood transplantation and, more importantly, the widespread adoption of haploidentical donor transplantation. Consequently, the potential advantages of autologous transplantation became limited to two main aspects: reducing early transplant-related mortality and providing a treatment option for patients with very high-risk disease or a frail biological condition [4].

Several factors contributed to the predominance of allogeneic transplantation following the initial wave of autologous transplantation in leukaemia. It became evident that mortality associated with autologous transplantation in patients with acute leukaemia was considerably higher than in patients with myeloma or lymphoma undergoing autologous transplantation. As a result, transplant centres adopted a substantially more cautious approach to autologous transplantation in acute leukaemia. Reported early mortality rates following autologous transplantation in acute leukaemia ranged from 6% to 9%, compared with only 1–4% in patients with myeloma or lymphoma [3].

Although the upper age limit for transplantation has increased substantially, frailty remains an important consideration even in autologous settings. At the same time, early mortality associated with allogeneic transplantation has declined significantly over recent decades [5,6].

Donor availability represented a major limitation in earlier decades. However, the development of umbilical cord blood transplantation, haploidentical transplantation, and other alternative donor strategies has markedly improved the availability of suitable donors [7].

The induction of a graft-versus-leukaemia (GVL) effect in autologous settings has not yet been successfully achieved, as demonstrated by the limited efficacy of agents such as IL-2 and cyclosporin A. Nevertheless, technological advances have revived interest in several older approaches and stimulated new attempts to induce GVL effects in autologous transplantation [8,9,10]. Recent developments, including magnetic sorting of CD96-positive cells, in vitro pulsing with oncolytic viruses, incorporation of PD-1 blockade, and strategies to activate NK cells, may offer promising opportunities in this field. The potential role of PD-1 blockade in enhancing GVL has been investigated using pembrolizumab maintenance following autologous transplantation in AML. However, the possible adverse effects of PD-1 inhibition, including thrombocytopenia, altered platelet function, and an increased thrombotic risk, must be carefully considered [11,12,13]. Further clinical experience is required before these approaches can be reliably evaluated in routine clinical practice.

In vivo purging of autologous stem cell grafts using combinations of cytosine arabinoside with etoposide, or ex vivo purging with mafosfamide, has been proposed as a potentially promising strategy. For many years, in vitro purging was not considered sufficiently reliable. Nevertheless, recent high-throughput approaches and screening methods have yielded encouraging preliminary results, although these strategies remain far from routine clinical use [14].

A potential disadvantage of autologous transplantation is the development of clonal haematopoiesis, similar to observations in patients with myeloma, lymphoma, or myelodysplastic syndromes [15,16].

Autologous transplantation is not completely free from complications such as thrombotic microangiopathy (TMA) and veno-occlusive disease (VOD). However, the incidence of these complications is considerably lower than that observed following allogeneic transplantation. TMA occurs less frequently in autologous settings, except in cases where high-dose platinum-based conditioning regimens are used. Similarly, the incidence of VOD after autologous transplantation has been reported to be below 5% [5,14].

Iron overload, particularly in patients with myelodysplastic syndromes or myelofibrosis receiving multiple transfusions, may also adversely affect stem cell mobilisation and harvesting [17].

For the vast majority of patients, allogeneic transplantation, owing to its potent anti-leukaemic and graft-versus-leukaemia effects, remains the preferred treatment strategy for achieving long-term remission or cure. This concept continues to be supported by contemporary clinical evidence, while the role of autologous transplantation in acute leukaemia has gradually diminished recently [14].

### 1.2. Recent Concepts Supporting the Re-Evaluation of Autologous Transplantation in Leukaemia

Recent advances in the molecular classification of AML, together with evolving concepts regarding the timing of allogeneic transplantation and the achievement of minimal residual disease (MRD) negativity, have renewed interest in the potential role of autologous transplantation in acute leukaemia. Other important facts are experiences with plasma cell leukaemia, special cases of APL and ALL, as well for autologous transplantations. Increasing evidence suggests that the timing of transplantation is critical for achieving deep molecular remission, rather than merely clinical or cytogenetic remission [4,14].

A predictive model based on MRD burden has identified a 0.05% threshold, below which patients have a significantly reduced risk of relapse, corresponding to an approximately 4.6-fold reduction in relative risk. These observations raise important questions about the role of MRD assessment in optimising autologous transplantation strategies for acute leukaemia. In particular, the impact of stem cell harvesting during deep molecular remission on relapse risk across leukaemia subtypes and biological subsets warrants further investigation [4,6].

It may therefore be hypothesised that the indications for autologous transplantation are not limited solely to elderly or frail patients, or to patients lacking suitable donors, but may also include selected biological or molecular subsets of acute leukaemia [6]. The introduction of alternative stem cell sources, including T-replete haploidentical bone marrow or peripheral blood transplantation, together with the increasing use of reduced-intensity conditioning (RIC), has expanded the feasibility of allogeneic transplantation to a substantial proportion of patients with acute leukaemia up to approximately 70 years of age [3,11].

Autologous stem cell transplantation (ASCT) as consolidation therapy in complete remission (CR) may still be a valuable option in selected clinical settings. Although ASCT is associated with a higher risk of relapse, it also offers several advantages, including lower non-relapse mortality, shorter hospitalisation, absence of acute graft-versus-host disease (GVHD), and improved long-term quality of life [6,14]. Furthermore, the introduction of intravenous busulfan (IV-BU) in combination with high-dose melphalan, together with improved MRD monitoring and post-transplant maintenance strategies, has renewed interest in autologous transplantation approaches [18].

Conversely, several innovative purging strategies have recently been explored. One approach involves the positive selection of normal haematopoietic stem cells using CD34 and/or CD133 markers. This strategy may be applicable in approximately 50% of AML cases, based on the absent or low expression of these markers at diagnosis and their relative stability at relapse. The method has been shown to achieve a 3–4 log reduction in tumour cell contamination while preserving a median recovery of approximately 50% of normal stem cell [6].

Another promising strategy involves ex vivo purging using oncolytic viruses such as myxoma virus (MYXV), which selectively eliminate leukaemic stem and progenitor cells from AML samples while sparing normal CD34-positive haematopoietic stem and progenitor cells capable of restoring haematopoiesis. In addition, magnetic-activated cell sorting using antibodies directed against AML-associated antigens, including CD96, has demonstrated encouraging results, achieving greater than 2-log depletion of target cells without impairing the viability or differentiation capacity of normal haematopoietic progenitor cells [6,14].

In vivo purging approaches have also shown promising results. Early intensification therapy with high-dose cytarabine and etoposide before stem cell collection has been associated with a significantly reduced risk of relapse, with reported disease-free survival rates of 68.8% versus 35.5% in patients who did not receive this strategy [8,10,11]. Furthermore, combinations of cryopreservation, hyperthermia, and the ether lipid ET-18-OCH3 have demonstrated at least 1-log depletion of AML blasts [12,14].

Recent advances in stem cell processing technologies, microbiome optimisation, and vaccination strategies targeting Wilms tumour gene 1 (WT-1) have also shown potential to induce anti-leukaemic immunoreactivity, even in autologous transplantation settings [12]. The selection of the most appropriate purging strategy is likely to depend on disease-specific characteristics, including minimal residual disease (MRD) burden and antigen expression profiles. It is also conceivable that microbiome modulation may further enhance the efficacy of these approaches [13,19,20].

Immunological interventions may additionally induce GVL-like anti-leukaemic effects through the administration of interferons or stem cell-derived natural killer (NK) cells [21,22]. Novel stem cell processing technologies may also modify the immunoreactive composition of harvested grafts, thereby potentially enhancing anti-leukaemic immune activity even in autologous transplantation settings [23].

In light of these developments, this review aims to evaluate the clinical settings in which autologous transplantation should be prioritised as an initial therapeutic strategy or considered a particularly valuable option, especially in selected subgroups of acute leukaemia where it may offer advantages over first-line allogeneic transplantation. In addition, this review explores clinical scenarios in which autologous transplantation may represent an important alternative to allogeneic transplantation, particularly when combined with maintenance therapies [6,18].

Efforts to induce a graft-versus-leukaemia (GVL) effect following autologous transplantation, despite decades of largely unsuccessful attempts, have recently regained attention. One notable example is the activation of natural killer (NK) cells combined with post-transplant administration of programmed death-1 (PD-1) inhibitors [13].

Recent studies suggest that selected subgroups of patients with acute leukaemia who achieve complete molecular remission may derive outcomes comparable to, or potentially better than, those achieved with allogeneic transplantation. This concept may be particularly relevant in frail patients, in whom autologous transplantation can be combined with maintenance therapies while avoiding the toxicities associated with allogeneic procedures. Consequently, survival outcomes in selected patients may equal or even exceed those observed after allogeneic transplantation. Ongoing developments in purging technologies and GVL-inducing strategies are likely to further the future balance between autologous and allogeneic transplantation approaches [18].

The following sections discuss the clinical indications and disease settings in which autologous transplantation may be considered in acute leukaemia.

In the setting of autologous transplantation for acute leukaemia, there is essentially no absolute upper age limit. However, frailty and biological fitness may represent more important limiting factors than chronological age alone. Even when allogeneic transplantation is considered the preferred therapeutic strategy, stem cell harvesting may still be advisable. Collected autologous stem cells may serve as a backup option in cases of graft failure or while awaiting identification of a suitable donor. CD34-positive selection and MRD quality considerations, along with intensification therapy prior to autologous transplant, might still be considered as important tools. Furthermore, in patients undergoing allogeneic transplantation near the upper biological or age-related limits of eligibility, treatment of relapse with autologous transplantation may occasionally represent a reasonable alternative approach [4,20].

## 2. Indications

### 2.1. Plasma Cell Leukaemia and Autologous Transplantation

Primary plasma cell leukaemia represents a strong indication for upfront autologous transplantation and is generally considered a standard component of first-line therapy. More recent data suggest that allogeneic transplantation does not provide a clear survival advantage over autologous transplantation in this setting. Maintenance therapy following transplantation remains an essential component of standard management [24,25,26,27].

### 2.2. Chronic Lymphocytic Leukaemia (CLL)

Autologous transplantation in CLL is associated with relatively high treatment-related mortality compared with its use in myeloma or other lymphomas. In addition, the introduction of highly effective targeted therapies capable of inducing deep remissions and improving survival has markedly reduced the role of autologous transplantation in CLL. Consequently, this approach is now considered largely obsolete, except in selected clinical situations, such as Richter transformation [28,29,30].

### 2.3. Acute Myeloid Leukaemia (AML)

Only a limited number of retrospective studies have compared outcomes following autologous transplantation with those achieved using alternative donor transplantation for remission consolidation in AML. Matched sibling and matched unrelated donor allogeneic stem cell transplantation remain the undisputed standard approaches in eligible patients [4,18,20].

The increasing availability of alternative stem cell sources, including T-cell-replete haploidentical bone marrow or peripheral blood transplantation, together with the wider use of reduced-intensity conditioning (RIC), has made allogeneic transplantation feasible for the majority of patients with acute leukaemia up to approximately 70 years of age. Nevertheless, autologous stem cell transplantation (ASCT) as consolidation therapy in complete remission may still represent an important alternative in selected cases. Although associated with a higher relapse risk, ASCT offers several advantages, including lower non-relapse mortality, the absence of graft-versus-host disease (GVHD), and improved long-term quality of life [6,20].

Recent studies have focused on identifying well-defined subsets of AML patients who may benefit from autologous transplantation, with outcomes comparable to, or in some cases even superior to, those achieved with allogeneic transplantation. In these subgroup analyses, achieving a deep MRD-negative first complete remission appears to be a key prerequisite [20,29].

Current evidence continues to support allogeneic transplantation as the preferred approach in poor-risk AML [14]. Consequently, interest in revisiting the role of autologous versus allogeneic transplantation has primarily focused on intermediate-risk AML [14]. In favourable-risk AML, both autologous and allogeneic transplantation have demonstrated 5-year relapse-free survival rates of approximately 60% [14,31,32].

In unselected intermediate-risk AML populations, 5-year survival following autologous transplantation was approximately 20% lower, mainly due to the higher incidence of relapse, which approached 20% in autologous settings [33]. However, data reported by Yoon et al. demonstrated that patients with intermediate-risk AML and favourable or absent adverse molecular alterations achieved a 5-year overall survival of 84% following autologous transplantation, which compared favourably with outcomes after allogeneic transplantation [33].

Similarly, patients with intermediate-risk cytogenetics and favourable molecular profiles demonstrated comparable overall survival following autologous or allogeneic transplantation, with reported survival rates of 62% and 66%, respectively. These findings suggest that autologous transplantation may be a valid, less toxic alternative for selected patient populations [29,32]. As additional molecular and clinical data become available, further subsets of intermediate-risk AML may emerge as potential candidates for autologous transplantation [29].

Intermediate-risk AML cases with favourable MRD characteristics, including RUNX1-RUNX1T1-positive disease (t(8;21)) with favourable quantitative MRD assessment, differential evaluation of IDH1 and IDH2 mutations, and the absence of FLT3 mutations, demonstrated outcomes comparable to those achieved with haploidentical transplantation. In contrast, haploidentical allogeneic transplantation appears to be more appropriate for intermediate-risk AML cases harbouring FLT3 mutations. The role of post-transplant anti-FLT3 maintenance therapy following autologous transplantation remains unclear [29,32,33].

The PETHEMA group published a large analysis demonstrating that AML patients without cytogenetic abnormalities and without FLT3-ITD mutations achieved superior response rates and survival following autologous transplantation compared with allogeneic transplantation. The authors therefore continue to consider autologous transplantation a valid alternative in selected AML patients, particularly in cases with an NPM1 mutation and FLT3-ITD negativity, in which the best outcomes were observed [34].

Additional studies have similarly suggested that selected patients within the intermediate-risk prognostic group may achieve superior outcomes following autologous transplantation compared with haploidentical allogeneic transplantation. However, patients harbouring FLT3 mutations experienced significantly inferior outcomes after autologous transplantation [35].

A Spanish multicentre study compared two molecular subgroups of AML patients with intermediate-risk cytogenetics: one group with favourable molecular features, including mutated NPM1 and biallelic CEBPA mutations, and another with unfavourable molecular characteristics, including non-mutated NPM1 and FLT3-ITD positivity. In the favourable molecular subgroup, outcomes following autologous transplantation were comparable to those achieved with allogeneic transplantation, with 5-year overall survival rates of 77% versus 70%, respectively. In contrast, outcomes in the unfavourable molecular subgroup remained poor, with approximately 40% 5-year overall survival, confirming that allogeneic transplantation remains the preferred standard approach in this setting [34].

Sula et al. reported excellent outcomes with autologous transplantation in core-binding factor (CBF)-positive AML. Although CBF-associated AML, particularly involving inv [16] or t(8;21), is generally associated with a relatively favourable prognosis even without transplantation, relapse remains a major clinical challenge. In contrast, patients with FLT3 mutations appear to derive greater benefit from haploidentical donor transplantation [35].

Overall, while allogeneic transplantation remains the preferred strategy for high-risk AML, autologous haematopoietic stem cell transplantation (auto-HSCT) may represent a valuable alternative for selected patients with favourable- or intermediate-risk molecular profiles, particularly when a matched donor is unavailable [36]. In patients experiencing a second AML relapse, repeated allogeneic transplantation is generally preferred over autologous transplantation [37].

Maintenance strategies after autologous transplantation remain an area of active investigation. Sorafenib maintenance has demonstrated clear benefit, particularly in FLT3-mutated AML. Additional data support the use of azacitidine and gilteritinib in FLT3-positive disease; however, most available evidence comes from allogeneic transplantation settings, and their role after autologous transplantation has not yet been adequately investigated. Nevertheless, maintenance therapy will likely become an increasingly important component of post-autologous transplantation management [14,29,36].

### 2.4. Acute Promyelocytic Leukaemia (APL)

APL medical treatment (all-transretinoic acid and arsenic-based) has recently achieved remission or cure in the vast majority of patients. However, in relapsed or refractory cases, depending on PML-RARA mutational results, an allogeneic bone marrow transplant should be considered. As an alternative approach, an autologous transplant might also be an option for selected patients.

Analysis of 294 patients demonstrated superior overall survival with autologous compared with allogeneic transplantation in APL patients undergoing transplantation in second complete remission. The reported 5-year overall survival was 75% following autologous transplantation versus 54% after allogeneic transplantation, mainly due to substantially lower treatment-related mortality (2% versus 30%) [38]. These findings have been incorporated into several international guidelines, including paediatric recommendations, particularly for patients in second remission in whom stem cells were collected during complete molecular remission [39,40,41,42,43].

### 2.5. AML: Summary

Although allogeneic transplantation remains the preferred strategy for high-risk AML, autologous transplantation represents a valuable alternative for selected patients with intermediate- or favourable-risk disease, particularly when a matched donor is unavailable. In general, autologous transplantation is not recommended in patients with adverse-risk cytogenetics [18,33,37].

In carefully selected molecular and clinical subgroups, outcomes following autologous transplantation may be comparable to, or occasionally better than, those achieved with allogeneic transplantation. Most studies have used intravenous busulfan-melphalan (IV-BU-Mel)-based conditioning regimens, which are associated with a lower incidence of veno-occlusive disease (VOD) compared with oral busulfan approaches. Less frequently used regimens include treosulfan-melphalan and high-dose cytarabine- and etoposide-based protocols [19,20,29].

Patients with favourable-risk AML and selected intermediate-risk AML lacking adverse cytogenetic abnormalities who achieve a first MRD-negative remission may derive substantial benefit from autologous transplantation. Core-binding factor (CBF) AML and CEBPA-mutated AML should also be carefully considered during therapeutic decision-making, as several studies suggest favourable outcomes even in second complete remission [44]. In contrast, patients with relapsed APL appear to achieve superior outcomes following autologous transplantation compared with allogeneic transplantation. High risk AML patients are not candidates for autologous transplantation modalities [11,27]. VOD and TMA risks are lower (less than 5%) in autologous settings, but they are not absent still, depending much on conditioning settings, like busuplhan dose and route of administration, heavily pretreated patients, pre-existing liver disease, etc. [5].

### 2.6. Acute Lymphoblastic Leukaemia (ALL)

Autologous stem cell transplantation (ASCT) is rarely used as frontline therapy in adult acute lymphoblastic leukaemia (ALL), particularly in standard-risk patients, because it has generally proven less effective than modern consolidation and maintenance chemotherapy strategies. In the final analysis of the International ALL Trial (MRC UKALL XII/ECOG E2993), patients assigned to autologous transplantation achieved a lower 5-year overall survival (OS) of 37% compared with 46% in patients receiving chemotherapy alone [45,46].

For standard-risk ALL, the greatest survival benefit is achieved with matched sibling allogeneic transplantation performed in first complete remission. Furthermore, recent advances in ALL treatment, including targeted therapies, bispecific antibodies, and cellular immunotherapies, have further reduced the role of autologous transplantation [45].

Nevertheless, autologous transplantation may still represent a reasonable alternative in selected situations, particularly when a matched donor is unavailable and in patients with favourable-risk disease who achieve MRD negativity. In these cases, the higher relapse risk associated with autologous transplantation must be balanced against the increased transplant-related mortality associated with allogeneic transplantation [46].

Several recent multicentre studies have suggested that autologous transplantation may remain a suitable therapeutic option in selected patients with Philadelphia chromosome-negative precursor T-cell ALL [46]. In addition, Giebel et al. analysed non-prospective data regarding autologous transplantation in Philadelphia chromosome-negative ALL patients older than 55 years and concluded that autologous transplantation may represent a feasible alternative approach with potential clinical benefit [47].

### 2.7. Chronic Myeloid Leukaemia (CML)

Autologous transplantation is not indicated as a primary therapeutic strategy in chronic myeloid leukaemia (CML), chronic neutrophilic leukaemia, or atypical CML, mainly because of the excellent efficacy of currently available targeted therapies. Indeed, the overall role of transplantation in CML has progressively diminished over recent decades. When transplantation is required, the preferred approach is allogeneic transplantation. Autologous transplantation is now used only exceptionally and is generally limited to highly selected elderly or frail patients, although remission durations are typically shorter than those achieved with allogeneic transplantation [48,49].

## 3. Discussion

Autologous transplantation in leukaemia was an important therapeutic option 20–30 years ago, mainly because of limited donor availability, stricter age restrictions, biological considerations, and lower early post-transplant mortality than allogeneic transplantation at that time [1,2,4,19]. Over subsequent decades, transplantation practices have evolved considerably, with the introduction of alternative donor sources (non-Twin/identical sibling, i.e., unrelated donors, umbilical cord, etc.) and haploidentical transplantation, along with substantial reductions in early mortality after allogeneic transplantation. Nevertheless, early mortality after autologous transplantation in leukaemia remains higher than that observed after autologous transplantation for myeloma or lymphoma [3,6]. In contrast, complications such as thrombotic microangiopathy (TMA) and veno-occlusive disease (VOD) remain less frequent in autologous settings [5].

Traditional approaches aimed at reducing relapse risk after autologous transplantation, including in vivo and in vitro stem cell purging and attempts to induce anti-leukaemic immune responses, have historically achieved limited success. In addition, secondary myelodysplastic syndromes (MDS) and clonal haematopoiesis have been reported following autologous transplantation, further restricting its use mainly to older or frail patients [9,15,16]. For many years, allogeneic transplantation therefore remained the preferred strategy because of its superior graft-versus-leukaemia (GVL) effects and lower relapse rates [7,8].

Reported disease-specific transplant-related (early) mortality (non-relapse mortality) following allogeneic transplantation for AML varies considerably according to disease status, donor type, conditioning regimen, and transplantation modality, ranging from approximately 9.4% to 27.5%. In contrast, early mortality after autologous transplantation in AML has generally ranged between 5% and 9%, while long-term non-relapse mortality at 10 years appears to remain around 5% [4,6,9,18,19,20,29,50,51].

More recently, this rather pessimistic view of autologous transplantation has begun to change. Novel in vitro purging and cell-selection strategies have been developed, although large prospective clinical trials are still lacking. In addition, activation of natural killer (NK) cells and administration of programmed death-1 (PD-1) inhibitors may potentially induce anti-leukaemic immune responses, although definitive clinical evidence remains limited [11,12,13,14,21,22,23]. Furthermore, several innovative cellular therapeutic approaches now utilise host-derived immune cells [50].

Perhaps the most important factors leading to the reconsideration of autologous transplantation have been advances in molecular classification and the recognition that achieving deep MRD-negative remission is critical across all transplantation settings in leukaemia [4,6,9,19,20,29]. On the other hand, the wide range average of early transplant mortality (very variable, individual, disease, MRD degree, conditioning, etc.) seems to be 6–11, with longer hospital stay and organ damage, whereas autologous transplant early cumulative mortality seems to be 1–4%, with shorter hospitalisation and less organ damage. An advantage of allogeneic transplant is the better standardised post-transplant maintenance therapy [20,32,33].

Importantly, certain disease entities remain strong indications for autologous transplantation. Plasma cell myeloma with plasma cell leukaemia remains one such example [24,25]. In addition, selected subgroups of AML, acute promyelocytic leukaemia (APL), and a small subset or age group of ALL patients may achieve acceptable, and in some settings even superior, outcomes with autologous transplantation [15,34,35]. In APL, particularly in second complete remission following molecular remission and stem cell collection, while MRD-negative, autologous transplantation has demonstrated particularly favourable outcomes. Long-term follow-up of these patients remains important because of the potential development of clonal haematopoiesis and secondary MDS after autologous transplantation [15,16].

Currently, no convincing evidence demonstrates that allogeneic transplantation from an alternative donor (haplo or umbilical cord) is superior to autologous transplantation in patients with good- or intermediate-risk AML who achieve undetectable MRD in first complete remission, particularly in the presence of favourable molecular alterations. Similar observations apply to patients with APL in second complete remission [31,32,33,34,35]. In plasma cell leukaemia and APL, the favourable outcomes associated with autologous transplantation may also partly reflect the use of less intensive conditioning regimens.

Finally, donor availability problems, advanced age, and impaired biological fitness remain important factors favouring consideration of autologous transplantation in selected patients. Still, it is worth considering that the age limit for being a candidate and eligible for RIC selection is moving upwards [20,29].

## Data Availability

No new data were created or analysed in this study.

## Abbreviations

The following abbreviations are used in this manuscript:

ALL	acute lymphoblastic leukaemia
AML	acute myeloid leukaemia
APL	acute promyelocytic leukaemia
Arhgef	Rho guanine exchange factor
ASCT	autologous stem cell transplantation
ATRA	all-trans retinoic acid
Bu-Cy	busulfan-cyclophosphamide conditioning regimen
BU	busulfan
BU-MEL	busulfan-melphalan conditioning regimen
CBF	core-binding factor
CEBPA	CCAAT/enhancer-binding protein alpha
CLL	chronic lymphocytic leukaemia
CML	chronic myeloid leukaemia
CR	complete remission
CR1, CR2	first and second complete remission
ET-18-OCH3	edelfosine, a cell-permeable reversible cytotoxic agent
FLT3	FMS-like tyrosine kinase 3
FLT3-ITD	FLT3 internal tandem duplication
FLU	fludarabine
FLU-MEL	fludarabine-melphalan conditioning regimen
GVHD	graft-versus-host disease
GVL	graft-versus-leukaemia effect
HSCT	haematopoietic stem cell transplantation
IDH1	isocitrate dehydrogenase 1
IDH2	isocitrate dehydrogenase 2
IL-2	interleukin 2
IRN	irinotecan-based treatment
IV	intravenous
MDS	myelodysplastic syndrome
MEL	melphalan
MRD	measurable residual disease
MYXV	myxoma virus
NK	natural killer
NPM1	nucleophosmin 1
OS	overall survival
PCR	polymerase chain reaction
PD-1	programmed death 1
PD-L1	programmed death ligand 1
PML-RARA	promyelocytic leukaemia-retinoic acid receptor alpha fusion gene
RIC	reduced-intensity conditioning
RUNX1-RUNX1T1	runt-related transcription factor 1–RUNX1 partner transcriptional co-repressor 1 fusion gene
TBI	total body irradiation
TMA	thrombotic microangiopathy
TRM	transplant-related mortality
VOD (SOS)	veno-occlusive disease (sinusoidal obstruction syndrome)
WT-1	Wilms tumour gene 1
